# Machine learning prediction of tuberculosis mortality: a comparative analysis of random survival forest and cox regression models

**DOI:** 10.1186/s12879-026-13109-9

**Published:** 2026-03-17

**Authors:** Azeez Adeboye, Osuji Georgeleen, Adelabu Olusesan, Noel Colin

**Affiliations:** 1 Gastrointestinal Research Unit, University of the Free State, Bloemfontein, South Africa; 2 Department of Computational Sciences, Faculty of Science and Agriculture, University of Fort Hare, Alice, Eastern Cape 5700 South Africa; 3 Department of Medical Microbiology, Faculty of Health Sciences, University of the Free State, Bloemfontein, South Africa; 4 Division of Gastrointestinal Surgery, Department of Surgery, Faculty of Health Sciences, University of the Free State, Bloemfontein, South Africa

**Keywords:** Random survival forest, Cox regression, Tuberculosis, Machine learning, Survival analysis

## Abstract

**Background:**

Survival analysis is widely used to predict time-to-event outcomes, with the Cox regression model being a standard approach. However, machine learning methods such as Random Survival Forests (RSF) can capture complex, non-linear relationships that traditional models may miss.

**Objective:**

This study compared the predictive performance of RSF and Cox regression in modelling tuberculosis (TB) mortality.

**Methods:**

We conducted a retrospective study of TB patients treated at the East London Central Clinic in South Africa. Patient data included demographic, clinical, and treatment-related variables. Model performance was evaluated using five metrics (C-index, Brier Score, Integrated Brier Score, Integrated Absolute Error, and Integrated Squared Error) along with time-dependent receiver operating characteristic (ROC) curves. Variable importance was assessed to identify key predictors.

**Results:**

The RSF model consistently outperformed the Cox model across all evaluation metrics. RSF achieved a higher integrated AUC (0.815 vs. 0.652) and lower prediction error (IBS = 0.235 vs. 0.261). Important predictors of mortality included age, sex, weight, and disease class, with RSF capturing their time-dependent effects more accurately. The cumulative case/dynamic control ROC curve showed the strongest predictive accuracy at 120 days (AUC = 0.856).

**Conclusion:**

RSF demonstrated superior predictive accuracy compared with Cox regression in modelling TB mortality. Its ability to account for non-linear and time-dependent effects makes it a potentially useful tool for improving risk prediction and guiding patient management in TB care.

**Clinical trial:**

Not applicable.

**Supplementary Information:**

The online version contains supplementary material available at 10.1186/s12879-026-13109-9.

## Introduction

Tuberculosis (TB) remains a leading cause of infectious disease mortality worldwide, responsible for more than 1.8 million deaths annually [1]. Despite advancements in treatment, TB continues to pose challenges due to its strong association with comorbidities such as HIV, diabetes, and malnutrition [2–5]. Also, TB is associated with a range of systemic metabolic, biochemical, haematological, and immunological disturbances that may influence disease progression and patient outcomes [4–11]. Accurate prediction of TB outcomes is therefore crucial for improving patient management and guiding resource allocation, particularly in high-burden regions.

Machine learning (ML) techniques have shown significant promise in predicting TB and drug resistance. One study achieved more than 80% high performance accuracy in predicting treatment success in TB patients using ML algorithms [12]. Another focused on feature selection algorithms for diagnosing multidrug-resistant TB, recommending random forest and support vector machine models for patient classification [13]. ML techniques are increasingly used to improve TB detection, diagnosis, and outcome prediction by enhancing image-based diagnostic accuracy [14]. Additionally, ML models have demonstrated strong performance in classifying TB based on clinical data [15–17], highlighting their potential in improving diagnosis and treatment.

Machine learning (ML) has been widely used to enhance TB diagnosis [18, 19] using clinical data, molecular biology information, and breathing acoustic signals, resulting in improved diagnostic accuracy [20–26]. Artificial neural networks (ANNs), particularly multilayer perceptron (MLP) models, are effective for detecting TB risk [18, 26–31]. Unlike prior studies that focused on TB diagnosis or drug resistance prediction, this study extends ML application to survival prediction, directly comparing RSF and Cox regression using real clinical data from South Africa. This study aims to evaluate the performance of RSF and Cox regression using patient data to identify key variables for predicting TB mortality in South Africa.

## Methods

### Study design and setting

This retrospective study used patient data extracted from both electronic records and TB treatment cards maintained at the East London Central Clinic-TB Unit, located in the Eastern Cape province of South Africa. The clinic adheres to standardized national TB treatment guidelines and maintains comprehensive, longitudinal patient records, which served as the primary data source for this study. Patients were eligible for inclusion if they have a confirmed diagnosis of TB recorded between January 2010 and December 2019, a documented date of diagnosis, and at least one recorded follow-up visit during treatment. These time boundaries were selected to ensure a sufficiently large dataset and to allow for adequate follow-up within a consistent clinical framework. Patients were excluded if key baseline clinical information was missing or incomplete and if the date of TB diagnosis or treatment initiation was not recorded. This criterion was essential to ensure data completeness, enable temporal analyses, and maintain the integrity of outcome measurements. These inclusion and exclusion criteria were deliberately chosen to balance the need for robust, analysable data with the desire to minimize selection bias. However, we acknowledge that excluding patients with incomplete records may limit the representativeness of the sample. Individuals who were lost to follow-up early or whose records were poorly maintained may be underrepresented. This could affect the generalizability of the findings to broader or more vulnerable TB populations.

### Data collection

Patient information was obtained from a specialized clinic that maintains records of drug-resistant TB, including demographic variables (sex, age, marital status, residence), clinical status (TB type, HIV status, diabetes status, weight), and treatment-related data such as ART use. In this study, the variables time, age, and weight were treated as continuous variables, as they were recorded using numerical values and summarized using means and standard deviations. The following variables were treated as categorical (binary) variables, as they consisted of two distinct categories: substance use (yes/no), sex (male/female), HIV status (positive/negative), disease class (dclass: pulmonary TB [PTB] / extrapulmonary TB [EPTB]), ART (yes/no), diabetes status (yes/no), residence (urban/rural), alcohol use (yes/no), and smoking (yes/no). Marital status was treated as a categorical nominal variable, with four categories: single, married, divorced/separated, and widowed. TB type (tbtype) was categorised as drug-resistant TB (DR-TB) and multidrug-resistant TB (MDR-TB). Cases with substantial missingness, defined as more than 20% missing across key variables were excluded from the analysis to preserve data quality and minimize bias. All patients had complete baseline data (complete-case analysis).

### Statistical analyses

We compared the predictive performance of RSF and Cox regression models. Five evaluation metrics were used: concordance index (C-index), Brier Score (BS), Integrated Brier Score (IBS), Integrated Absolute Error (IAE), and Integrated Squared Error (ISE). Cumulative/Dynamic Time-dependent ROC analysis was used to assess predictive accuracy at different follow-up intervals proposed by Heagerty and Zheng (2005) [33].

#### Concordance index (C-index)

Let ${\delta}_{i}=1$ and ${\delta}_{j}=1$ represent the number of deaths observed in both samples, and ${T}_{i}={X}_{i}={X}_{j}={T}_{j}$ represents the observed survival time. The C-index metric value can be expressed as.

$$\mathrm{C-index}=\frac{CC+0.5\times{PC}}{\sum_{i,j}n{p}_{ij}\:({X}_{i},{\delta}_{i},{X}_{j},{\delta}_{j})}$$

where ${\delta}_{i}$ represents the survival status of sample $i$, ${X}_{i}$ is the observed survival time, CC is the complete concordance and is defined as $\sum_{i,j}I\left(\mathrm{sign}\left({Y}_{i},{Y}_{j}\right)=\mathrm{csign}\left(X,{X}_{j}\right)|n{p}_{ij}=1\right),$ PC is the partial concordance, and $n{p}_{ij}=\left({X}_{i},{\delta}_{i},{X}_{j},{\delta}_{j}\right)$. The details can be found in [32]. However, the larger the C-index is, the better the prediction accuracy of the model.

#### Brier score (BS)

The BS is another metric used for evaluating accuracy prediction performance [33] and can be expressed as follows.

$$\begin{aligned}&BS\left({t}^{*}\right)=\frac{1}{N}\cr&\sum_{i=1}^{N}\left[\begin{aligned}&\frac{{\left(\widehat{S}\left({t}^{*}|{z}_{i}\right)\right)}^{2}}{\widehat{G}\left({X}_{i}\right)}.I\left({X}_{i}<{t}^{*},{\delta}_{i}=1\right)\cr&+\frac{\left(1-{\left(\widehat{S}\left({t}^{*}|{z}_{i}\right)\right)}^{2}\right)}{\widehat{G}\left({t}^{*}\right)}.I\left({X}_{i}\ge{t}^{*}\right)\end{aligned}\right]\end{aligned}$$

where ${t}^{*}$ represents the calculated BS at a given time point with sample size $N$; ${z}_{i}$ represents sample covariate $i$; $\widehat{S}\left(.\right)$ is the model-predicted survival function; and $\widehat{G}\left(.\right)$ is the censoring survival function. The BS value is essentially a square prediction error based on inverse probability censoring weighting (IPCW). The smaller the value is, the better the prediction performance of the model.

#### Integrated brier score (IBS)

The IBS focuses primarily on residuals across all observed time points. When the time of interest extends beyond a specific time point, IBS can offer more comprehensive information than BS. The IBS can be expressed as.

$$IBS=\frac{1}{\genfrac{}{}{0pt}{}{\mathrm{max}}{i}\left({X}_{i}\right)}\underset{0}{\overset{\genfrac{}{}{0pt}{}{\mathrm{max}}{i}\left({X}_{i}\right)}{\int}}BS\left(t\right)dt$$

Like for the BS criteria, a lower IBS value generally indicates a better model prediction.

#### Integrated absolute error (IAE)

The IAE is sometimes employed to assess the difference between the estimated survival function and the true survival function. It can be expressed as

$$IAE=\int|S\left(t\right)-\widehat{S}\left(t|X\right)|dt$$

where $S\left(t\right)$ and $\widehat{S}\left(t\right)$ are the true survival function and predicted survival function, respectively, and X is the covariate of the training set. A lower IAE value generally indicates a better model prediction.

#### Integrated square error (ISE)

The ISE is similar to the IAE in computation. It is generally expressed as

$$ISE=\int{(S({t})-\widehat{S}({t|X}))}^{2}dt$$

where $S\left(t\right)$ and $\widehat{S}\left(t\right)$ are represented as above. A lower ISE indicates a better model prediction.

### The Cox PH model

Let ${P}_{n}=\{{T}_{i},{\delta}_{i},{x}_{i},{y}_{i};i=1,\dots{n}\}$ be the observed data, where ${T}_{i}=\mathrm{min}\left({T}_{i}^{*},{C}_{i}\right)$ is the event time observed for subject $i=1,\dots{n}$, ${C}_{i}$ represents the censoring time, ${T}_{i}^{*}$ represents the true event time, and ${\delta}_{i}=I\left({T}_{i}^{*}\le{C}_{i}\right)$ denotes the event indicator. The Cox proportional hazards model, commonly used in survival analysis and machine learning for time-to-event data, is expressed mathematically as follows:

$$h\left(t,X\right)={h}_{0}\left(t\right)\mathrm{exp}\left({\beta}_{1}{X}_{1}+\dots+{\beta}_{p}{X}_{p}\right)$$

where $h\left(t,X\right)$ is the hazard function at time $t$ for a given set of covariates X; ${h}_{0}\left(t\right)$ is the baseline hazard function, representing the hazard at time $t$ when all covariates are zero; and ${\beta}_{1}+\dots+{\beta}_{p}$ are the coefficients associated with the covariates ${X}_{1}+\dots+{X}_{p}$, representing the log hazard ratio for each covariate. The hazard function $h\left(t,X\right)$ describes the instantaneous risk of an event (e.g., death) occurring at time $\left(t\right)$, given a set of covariates X. The Cox model assumes that the hazard ratio for any two individuals is constant over time, which is a proportional hazards assumption. Right censoring was applied to account for individuals whose event time is unknown beyond the last follow-up.

### The random survival forests (RSF)

RSF is an extension of the random forest algorithm, an ensemble tree-based technique designed to analyse right-censored survival data [34, 35]. The construction of survival trees involves recursively partitioning the covariate space through binary splits, creating groups of subjects with similar survival outcomes [36]. The RSF algorithm is an extension of the traditional random forest algorithm for survival analysis. The RSF algorithm is an ensemble method that combines the predictions of multiple survival trees. The RSF algorithm description steps are as follows:

1. B bootstrap samples were generated from the original dataset, with each sample excluding approximately 37% of the data; these samples were referred to as out-of-bag (OOB) data.
2. A survival tree was constructed for each bootstrap sample. At each tree node, p candidate variables are randomly chosen, and the node is split using the variable that maximizes the survival difference between the resulting daughter nodes.
3. Expand each tree to its full size, adhering to the condition that a terminal node must contain no fewer than d_0 > 0 unique deaths.
4. Compute a cumulative hazard function (CHF) for each tree and then average them to obtain the ensemble CHF.
5. Evaluate the prediction error for the ensemble CHF using the OOB data.

The RSF algorithm can be expressed as follows:

#### Individual tree prediction of RSF

$${H}_{i}\left(t\right)=-\sum_{j-1}^{m}{\theta}_{ij}I\left\{t\in{R}_{ij}\right\}$$

where $m$ is the number of terminal nodes in the tree, ${\theta}_{ij}$ is the cumulative hazard contribution for the jth terminal node of the ith tree, and ${R}_{ij}$ is the region (subset of feature space) corresponding to the jth terminal node.

#### Trees combined with the forest

$$H\left(t\right)=\frac{1}{B}\sum_{i-1}^{B}{H}_{i}\left(t\right)$$

where $B$ is the number of trees in the forest. $S\left(t\right)$ is obtained by exponentiating the negative of the cumulative hazard function.

### RSF prediction error

#### OOB prediction error

The out-of-bag (OOB) ensemble CHF was employed to establish the predicted outcome because this value is derived from OOB data. The OOB value was used to estimate the C-index, and the OOB error rate was calculated. We use ${t}_{1}^{0},\dots,{t}_{m}^{0}$ represent preselected distinct time points ${t}_{1},\dots,{t}_{N}$. To rank two cases, $i$ and $j$, we assert that case $i$ has a predicted outcome that is less favourable than that of case $j$ if

$$\sum_{l-1}^{m}{H}_{e}^{**}\left({t}_{l}^{0}|{x}_{i}\right)>\sum_{l-1}^{m}{H}_{e}^{**}\left({t}_{l}^{0}|{x}_{j}\right)$$

This rule was applied to compute the OOB estimate by the C-index [37]. The OOB prediction error ($PE$) is defined as 1 – C and ranges from $0\le{PE}\le1$, and a $PE=0.5$ suggests that the prediction performance is no better than random guessing [38].

### Variable importance

The RSF offers meaningful insights into the factors that play a significant role in survival prediction by providing a variable importance (VIMP) measure for each variable. The VIMP was determined using OOB observations that were excluded from the tree-building process. A VIMP value of zero or less at a given time point signifies that the corresponding variable lacks predictive power for survival within that specific timeframe for the patient’s treatment period. Notably, the VIMP can fluctuate over the treatment period for various covariates, providing clinicians with crucial information about how the significance of these factors evolves as a patient progresses beyond baseline time points. This dynamic perspective enhances the interpretability of RSF models in the context of changing survival outcomes [39].

### Assessing predictive performance

The model’s predictive performance was evaluated using receiver operating characteristic (ROC) curves for TB treatment outcome using a time-dependent ROC curve that changes over time to provide an adequate description of the model’s prediction. The prediction performance was measured as a function of time by the curves for cumulative case control ROC using sensitivity and specificity at time $t$ at a threshold $c$ defined as:

$${\mathrm{sensitivity}}^{CC}\left(c,t\right)=P\left({S}_{i}>c|{T}_{i}\le{t}\right)$$

$${\mathrm{specificity}}^{D}\left(c,t\right)=P\left({S}_{i}\le{c}|{T}_{i}>t\right)$$

This approach assesses the prediction performance of the risk (cumulative incidence) of events over the specified ‘*t*’-year period, with true positives being those who have a marker value higher than the threshold ‘*c*.’ On the other hand, incident cases control the ROC curve using sensitivity and specificity at time $t$ at a threshold $c$ defined as:

$${\mathrm{sensitivity}}^{IC}\left(c,t\right)=P\left({S}_{i}>c|{T}_{i}=t\right)$$

$${\mathrm{specificity}}^{D}\left(c,t\right)=P\left({S}_{i}\le{c}|{T}_{i}>t\right)$$

However, the dynamic specificity of these methods remains constant. This definition involves partitioning the risk set at time ‘$t$’ (comprising individuals with ${T}_{i}\ge{t}$) into cases and controls [32, 40].

## Results

### TB patient characteristics

A total of 910 participants were included in the study. The mean age was 37.7 ± 15.0 years, with a mean weight of 51.7 ± 8.9 kg. The average duration on tuberculosis (TB) treatment was 129.6 ± 141.9 days. Females comprised 63.7% of the cohort, while 36.3% were male. HIV infection was present in 44.0% of participants, and 23.1% were on antiretroviral therapy (ART). Substance use was reported by 11.6%, alcohol use by 56.0%, and smoking by 25.1%. Diabetes was documented in 41.5% of the population. Most participants (79.7%) had pulmonary TB (PTB), and 20.3% had extrapulmonary TB (EPTB). Drug-resistant TB (DR-TB) accounted for 77.0% of cases, while 23.0% had multidrug-resistant TB (MDR-TB). In terms of marital status, 43.7% were single, 27.1% were married, and 29.0% were divorced or separated. Urban residents comprised 54.3% of the sample, compared to 45.7% from rural areas. At the end of the follow-up period, 87.0% of participants were alive, and 13.0% had died (Table 1).

**Table 1:** Baseline characteristics of the study population (*N* = 910)

Variable	Category	*N* (%)
Time (days)	Median ± SD	88.5 ± 155.8
Age (years)	Mean ± SD	37.7 ± 15.0
Weight (kg)	Mean ± SD	51.7 ± 8.9
Substance use	No	804 (88.4%)
	Yes	106 (11.6%)
Sex	Female	580 (63.7%)
	Male	330 (36.3%)
Marital status	Single	398 (43.7%)
	Married	247 (27.1%)
	Divorced/separated	264 (29.0%)
	Widow	1 (0.1%)
Residence	Rural	416 (45.7%)
	Urban	494 (54.3%)
Disease class (dclass)	EPTB	185 (20.3%)
	PTB	725 (79.7%)
TB type (tbtype)	DR-TB	701 (77.0%)
	MDR-TB	209 (23.0%)
HIV status	Positive	400 (44.0%)
	Negative	510 (56.0%)
ART	No	700 (76.9%)
	Yes	210 (23.1%)
Diabetes	No	532 (58.5%)
	Yes	378 (41.5%)
Alcohol	No	400 (44.0%)
	Yes	510 (56.0%)
Smoking	No	682 (74.9%)
	Yes	228 (25.1%)
Status	Alive	791 (87.0%)
	Died	119 (13.0%)

Note: ART- Antiretroviral Therapy; EPTB – Extra-Pulmonary Tuberculosis; PTB – Pulmonary Tuberculosis; MDR-TB – Multidrug-Resistant Tuberculosis; DR-TB – Drug-Resistant Tuberculosis; substance use: - defined as the use of hard drugs or non-medical substances during or before TB treatment initiation

### Model performance

#### Evaluation metrics and risk score prediction values for the RSF and Cox models

**Fig. 1:**
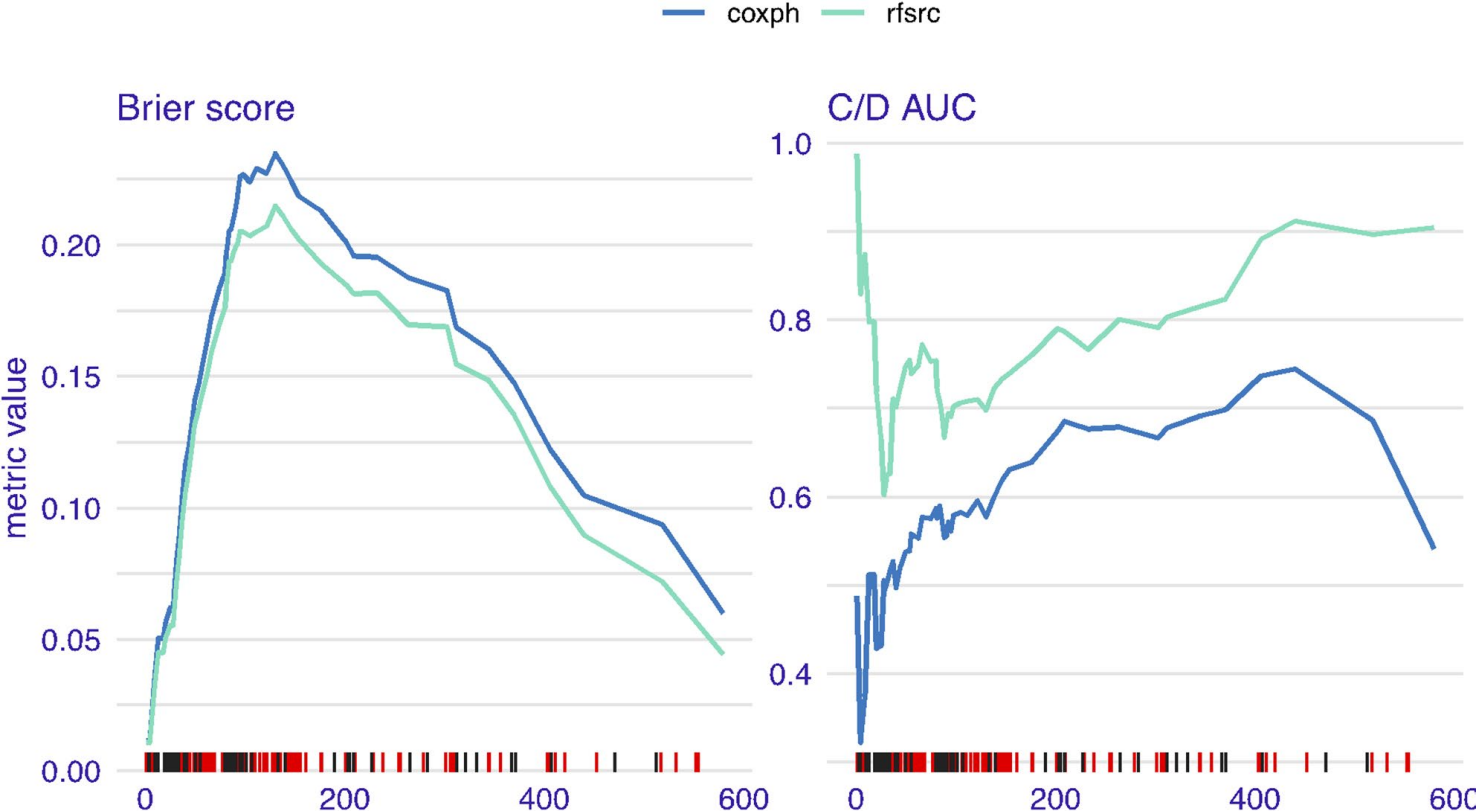
Model performance metrics for the Cox model (coxph) and RSF model using using the Brier score and Cumulative case/dynamic control (C/D) AUC. Coxph refers to Cox Proportional Hazards Model and rfsrc refers to Random Survival Forest

Both RSF and Cox regression models were trained on a subset of the data and tested on the remaining patients. Across all evaluation metrics, the RSF model consistently outperformed the Cox model. For instance, the RSF achieved a higher C-index, indicating better ability to distinguish survival times, and lower Brier scores, showing more accurate predictions. The Integrated Brier Score (IBS) was 0.252 for RSF compared with 0.277 for Cox regression summarised in Table 2 (also see Figure S1), and the integrated AUC was substantially higher for RSF (0.815 vs. 0.652) in Fig. 1. These results confirm that RSF provided better discrimination, calibration, and accuracy.

**Table 2:** Evaluation metrics for two different survival analysis models

Model metrics	Standard model input methods		Non-Standard model input methods	
	RSF model	Cox model	RSF model	Cox model
C index	0.518	0.507	0.518	0.507
Brier Score	0.230	0.254	0.230	0.254
IBS	0.252	0.277	0.259	0.269
IAE	180.298	180.560	23.206	29.629
ISE	68.311	68.451	1.258	1.945

Note: Standard model input methods- use original input features without additional transformations; Non-standard model input methods- apply alternative preprocessing, transformations, or feature engineering

#### Time-dependent variable importance influencing the models’ predictions (global explanation)

**Fig. 2:**
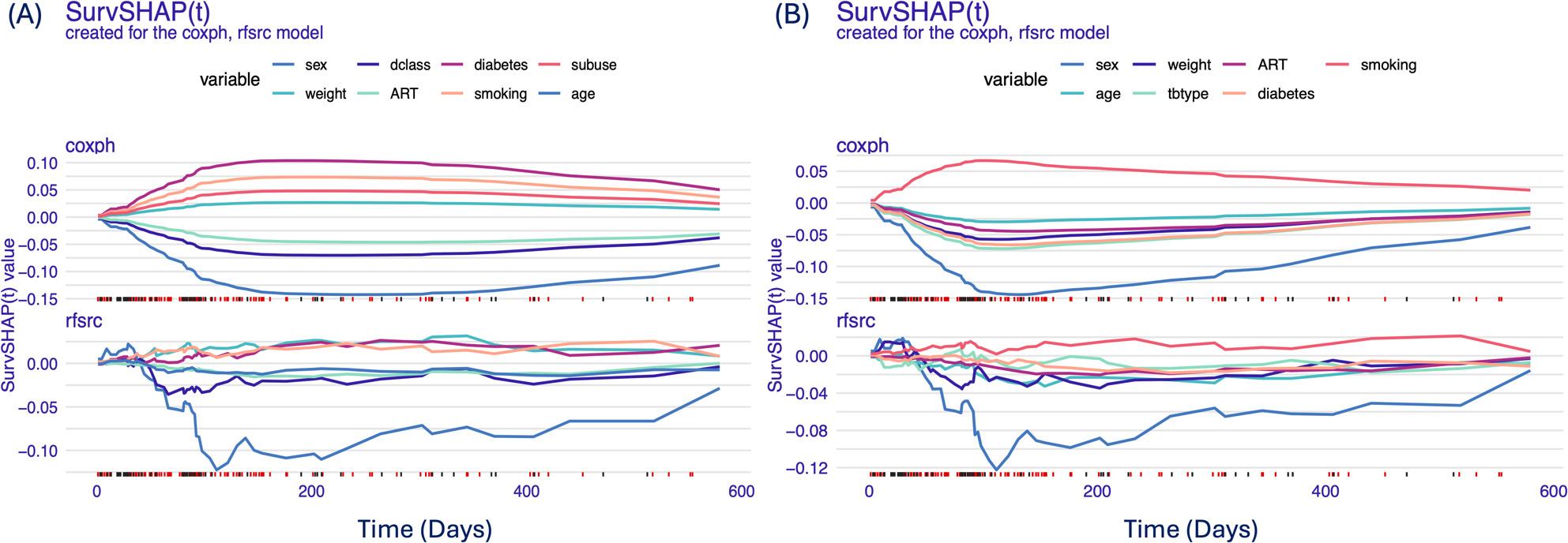
Time-dependent variable importance influencing the models’ predictions (global (upper) and local (lower) accuracy explanations)

**Fig. 3:**
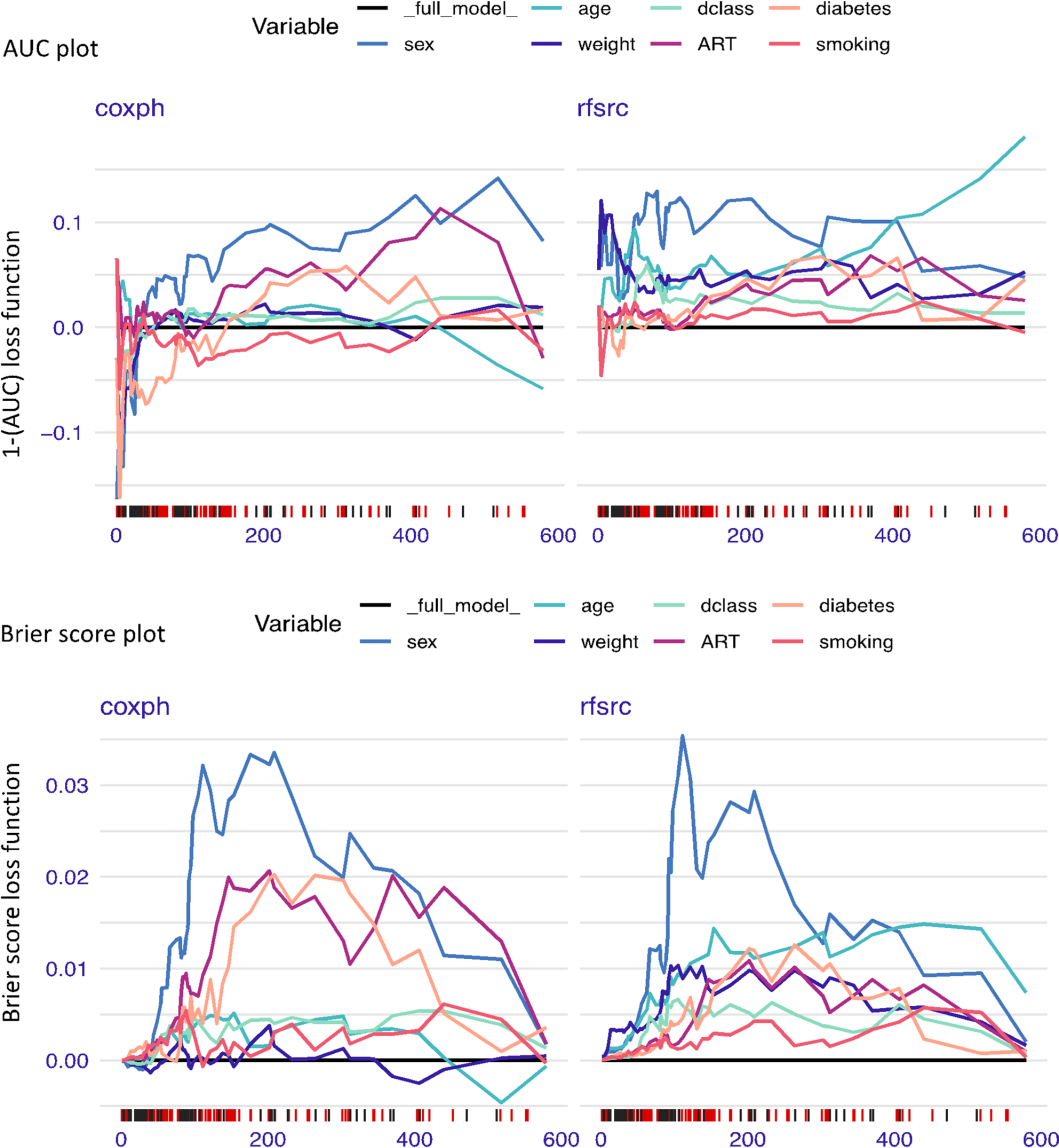
Variable importance determined using the loss function to ensure that this observation is consistent

Figure 2 displays the survival black-box model generated using the Shapley additive explanations function [41] for a specific prediction. Analysis of variable importance revealed that sex, disease class, weight, and age were among the most influential predictors of TB survival (Fig. 2). The RSF model was able to capture time-dependent changes in variable effects more accurately than the Cox model, highlighting its flexibility in handling dynamic survival patterns (Fig. 3). Loss function was applied to ensure that the observations were consistent (Fig. 3). In the AUC plot and Brier score plot, the results were consistent in both the RSF and Cox models for the influence of time-dependent variables on the models’ predictions.

### Minimal depth variable interaction

**Fig. 4:**
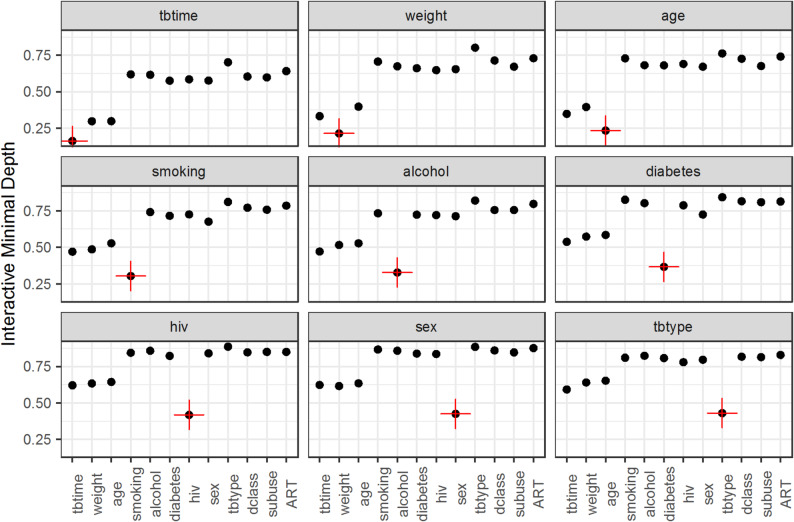
Minimal depth variable interaction plot for six variables of interest. Higher values indicate a lower association with the target variable, denoted in red

The RSF model was trained using 16 predictor variables and an ensemble of 500 trees, with an average of about seven terminal nodes per tree. The minimum node size was set to 15, and four variables were randomly selected at each split. Model splitting was guided by the log-rank test, and resampling was performed without replacement (Table S1). Minimal depth analysis provided insight, ranking the variables by their proximity to the root of the survival trees. The approach consistently highlighted the same set of key predictors. Together, these findings demonstrate that RSF not only produced accurate survival estimates but also offered meaningful insights into the relative influence and interactions of clinical variables over time (Fig. 4).

#### Cumulative/dynamic time-dependent ROC analysis for survival prediction

**Fig. 5:**
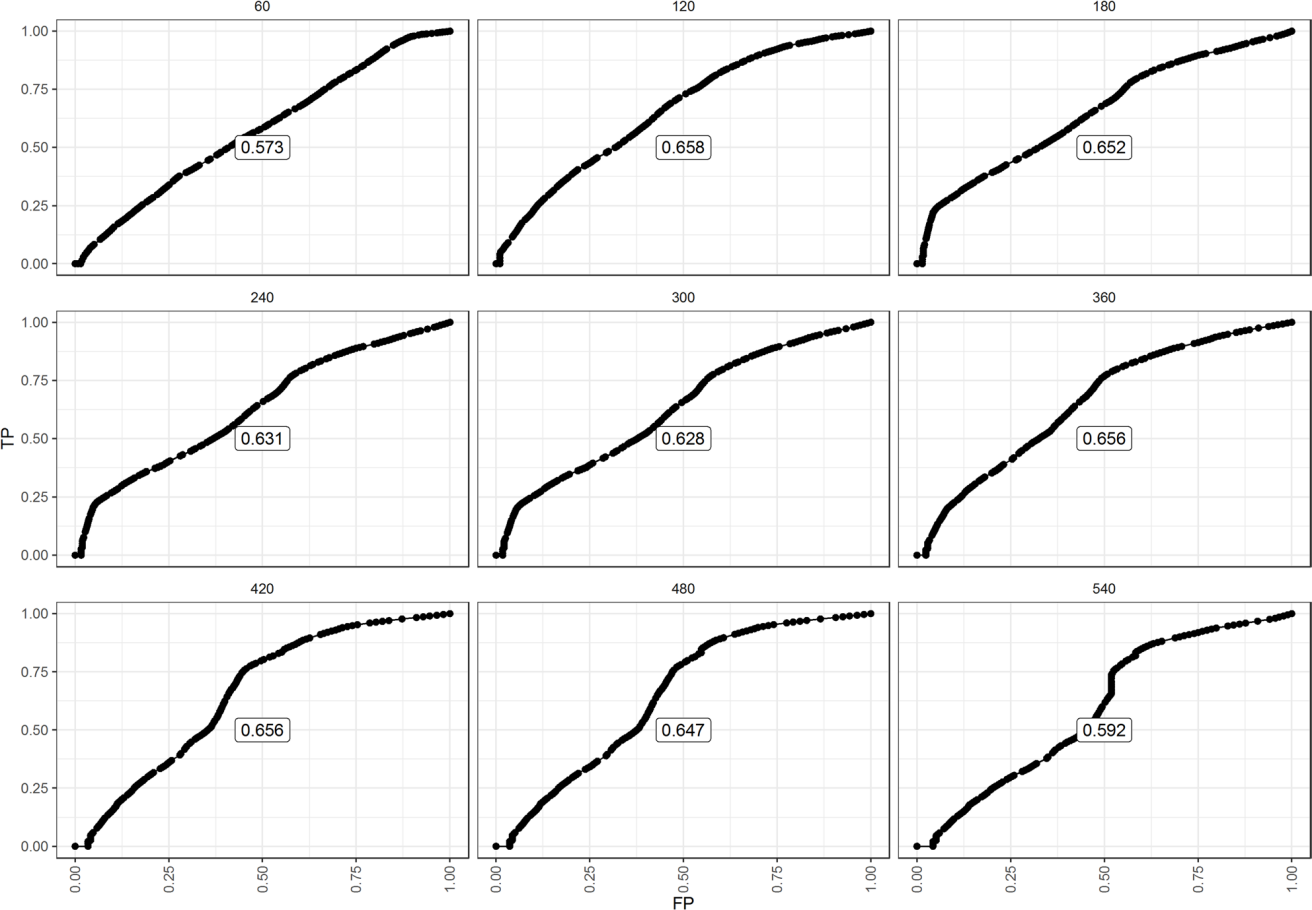
The cumulative case/dynamic control ROC plot for the survival prediction of TB patients experiencing the event at 120 days. TP (True Positive) and FP (False Positive)

**Fig. 6:**
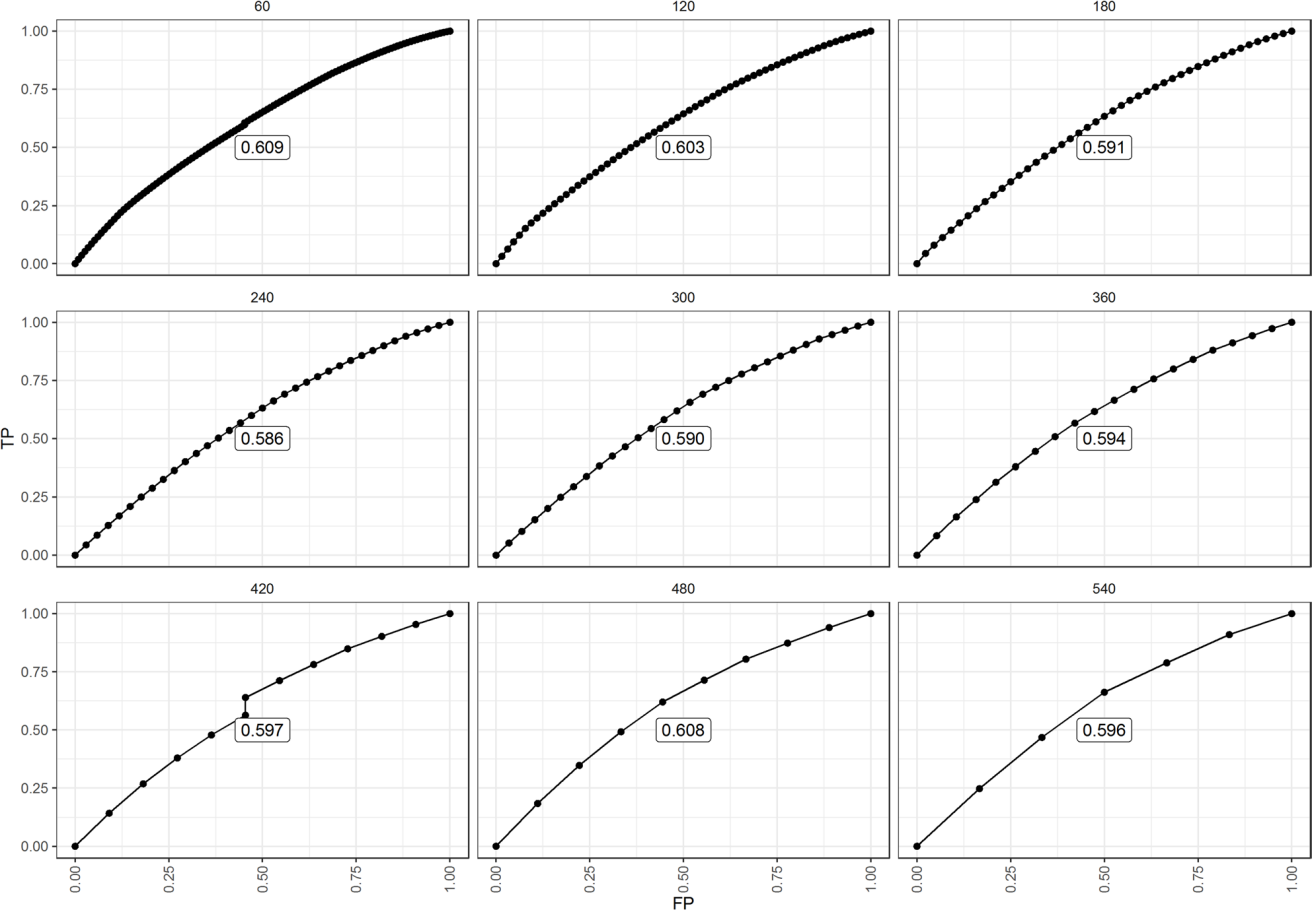
The incident case/dynamic control ROC plot for examining how long the time-zero variable is relevant in predicting survival events. TP (True Positive) and FP (False Positive)

The cumulative case/dynamic control ROC analysis showed that RSF achieved its best predictive performance at 120 days (AUC = 0.652, $\left(t\ge360\right)$). This suggests that RSF is particularly effective for early prediction of mortality risk, while maintaining stable performance throughout follow-up (Fig. 5). The incident cumulative case/dynamic control ROC1 risk prediction ROC curve of TB patients at time t at a threshold presented in Fig. 6 notably shows that while early risk predictions performed well, their accuracy declined over time likely because fewer high-risk individuals remained under observation, highlighting that the initial risk score became less informative for long-term survivors.

## Discussion

In this study, using patient information obtained from a specialized clinic that maintains records of drug-resistant tuberculosis, we compared the performance of the standard Cox model with ML-based RSF models for prognostic prediction in TB patients. Our results showed that the RSF model consistently and significantly outperformed the Cox model. Specifically, the RSF achieved a higher concordance index (C-index = 0.518) for both standard and nonstandard model inputs. These findings are consistent with previous studies reporting that RSF models often perform slightly better than Cox models in terms of discrimination [42–46]. Moreover, the RSF model demonstrated superior predictive accuracy, with a higher case/dynamic (C/D) AUC of 0.815 compared to 0.652 for the Cox model. This result is in line with Rahman et al. (2023) [47], who found that RSF (AUC = 0.839) outperformed Cox (AUC = 0.823) in predicting survival for patients with oesophageal cancer.

The RSF model also yielded lower prediction errors than the other models, and its algorithm provides a better alternative for estimating the survival probability among TB patients. The findings were consistent with a study by Sun et al. (2023) [42]. The BS, a measure of prediction accuracy, was consistently lower for RSF than for other models, which similar to findings by Liu et al. (2021) [48]. The RSF achieved lower Integrated Brier Score (IBS), Integrated Absolute Error (IAE), and Integrated Squared Error (ISE), further confirming its superior performance. These results agree with prior studies by Rahman et al. (2023) [47]. We also illustrated the application of RSF in evaluating the prediction accuracy of survival outcomes using RSF landmarking. Variable importance (VIMP) analysis identified age and other baseline covariates as strong predictors of TB outcomes. This finding aligns with earlier studies [49, 50], where age and related factors were strongly associated with biomarker predictions. Importantly, VIMP plots revealed dynamic relationships between predictors and survival outcome, emphasizing their changing influence over time.

Our analysis of cumulative case/dynamic control ROC curves demonstrated that RSF effectively predicts TB survival at different time points. The 120-day ROC curve achieved the highest predictive performance, likely due to the accumulation of events by this time. In 180–240 days, the AUC was decreasing, suggesting that predictive accuracy is maintained by high-risk individuals who experienced mortality early, but increases thereafter. This trend is consistent with findings in [51], who reported stable predictive performance across time, though it contrasts with [52], who observed poor prediction at 12 months.

In contrast, the incident case/dynamic control ROC1 curves showed a sharper decline in predictive accuracy over time. Because ROC1 considers only individuals at risk at each time point, the number of data points decreases, leading to less stable estimates. This reduction suggests that the predictive value of baseline risk scores weakens for patients who survive longer, as their risk profiles evolve. The final plots (Fig. 6) contained fewer data points, indicating reduced TB mortality risk among long-term survivors, which consistent with the notion that individuals surviving longer exhibit a decreasing hazard rate over time, reflecting improved survival probability conditional on prior survival. Collectively, these results highlight the importance of incorporating temporal dynamics when evaluating prognostic models.

While our primary aim was to compare the predictive performance of Cox and Random Survival Forest (RSF) models in identifying key clinical variables, the findings have potential implications for clinical practice. RSF models could support risk stratification efforts in TB management by identifying patients at higher risk of mortality, potentially enabling more personalized monitoring or intervention strategies. Its ability to model nonlinear interactions without assuming proportional hazards may provide a more flexible framework for developing decision-support tools, especially in settings with complex clinical features. While our study did not implement a clinical risk, future work could build on these results to develop individualized prediction tools integrated into clinical workflows. Importantly, both models identified consistent clinical predictors such as sex, age, ART status, weight, and comorbidities which align with known TB risk factors and could guide prioritization in patient care.

## Conclusion

This study compared the Random Survival Forest (RSF) model with the Cox proportional hazards model for predicting mortality among TB patients in South Africa. The findings demonstrated that RSF achieved superior predictive performance, including higher discrimination, improved calibration, and lower prediction error. By identifying key risk factors such as age, sex, weight, and disease class, and by accommodating their time-dependent effects, RSF provides a more flexible and clinically relevant approach to survival modelling than the Cox model. However, RSF represents a promising complementary tool for survival prediction in TB care, particularly in resource-limited settings where improved risk stratification may support better clinical decision-making and patient outcomes.

Despite these strengths, several limitations should be acknowledged. First, the RSF models employed the log-rank split rule, which relies on the proportional hazard’s assumption. When hazard functions cross, violations of this assumption may occur, potentially undermining predictive performance. Alternative splitting rules, such as those based on the integrated absolute difference between daughter node survival functions, may be proposed and may offer greater robustness. Second, RSF models are inherently less interpretable than Cox proportional hazards models, as the effects of individual predictors cannot be directly quantified and typically require supplementary tools such as variable importance measures or partial dependence plots. The analysis was based on data from a single TB clinic in the Eastern Cape, South Africa, and excluded patients with incomplete records, which may limit the generalizability of our findings to broader TB populations or other clinical settings. Future research should explore more robust splitting methods and validate RSF in larger, multi-center datasets to improve interpretability and confirm the generalizability of these findings.

## Supplementary Information

Below is the link to the electronic supplementary material.

Supplementary Material 1

## Data Availability

The dataset used in this study is available on request from the corresponding author. The R codes can be found: https://github.com/azizadeboye/RSF-vs-Cox-model.

## Abbreviations

ANN: Artificial Neural Network
ART: Antiretroviral Therapy
AUC: Area Under the Curve
BS: Brier Score
C-index: Concordance Index
C/D AUC: Case/Dynamic Area Under the Curve
CHF: Cumulative Hazard Function
DR-TB: Drug–Resistant Tuberculosis
EPTB: Extrapulmonary Tuberculosis
IAE: Integrated Absolute Error
IBS: Integrated Brier Score
IPCW: Inverse Probability of Censoring Weighting
ISE: Integrated Squared Error
MDR-TB: Multidrug–Resistant Tuberculosis
ML: Machine Learning
MLP: Multilayer Perceptron
OOB: Out–of–Bag
PH: Proportional Hazards
PTB: Pulmonary Tuberculosis
ROC: Receiver Operating Characteristic
RSF: Random Survival Forest
TB: Tuberculosis
VIMP: Variable Importance
