# Supplementary material for: Machine learning prediction of tuberculosis mortality: a comparative analysis of random survival forest and cox regression models
Source: BMC Infect Dis. 2026 Mar 17;26:829. doi: 10.1186/s12879-026-13109-9 (PMC13107631; doi:10.1186/s12879-026-13109-9)
Supplement: Supplementary file 1 — Supplementary Material 1 [file 12879_2026_13109_MOESM1_ESM.docx]

The RSF model is superior to the Cox model. Based on the metrics, the RSF model appears to be a better-performing model compared to the Cox model (Figure S1).

**Figure S1:** Scalar metrics in the form of bar plots to check Model performance for the Cox model and the RSF model.

**Table S1: Random Survival Forest (RSF) training configuration, hyperparameters, and out-of-bag performance metrics**

| Sample size (training set) | 728 |
| --- | --- |
| Number of deaths: | 89 |
| Number of trees: | 500 |
| Forest terminal node size: | 15 |
| Average no. of terminal nodes: | 7.13 |
| No. of variables tried at each split: | 4 |
| Total no. of variables: | 13 |
| Resampling used to grow trees: | swor |
| Resample size used to grow trees: | 89 |
| Analysis: | RSF |
| Family: | surv |
| Splitting rule: | logrank *random* |
| Number of random split points: | 10 |
| (OOB) CRPS: | 0.14842084 |
| (OOB) Requested performance error: | 0.5002207 |

[1] Michail, Loulakis., Charalambos, Makridakis. (2023). A new approach to generalisation error of machine learning algorithms: Estimates and convergence. arXiv.org, doi: 10.48550/arXiv.2306.13784
